# Bird-feeder cleaning lowers disease severity in rural but not urban birds

**DOI:** 10.1038/s41598-021-92117-y

**Published:** 2021-06-18

**Authors:** Laren Schaper, Pierce Hutton, Kevin J. McGraw

**Affiliations:** 1 Barrett The Honors College, Arizona State University, Tempe, AZ 85287 USA; 2 School of Life Sciences, Arizona State University, Tempe, AZ 85287 USA

**Keywords:** Ecological epidemiology, Gastrointestinal diseases, Ecology, Evolution, Zoology

## Abstract

Animals inhabiting urban areas often experience elevated disease threats, putatively due to factors such as increased population density and horizontal transmission or decreased immunity (e.g. due to nutrition, pollution, stress). However, for animals that take advantage of human food subsidies, like feeder-visiting birds, an additional mechanism may include exposure to contaminated feeders as fomites. There are some published associations between bird feeder presence/density and avian disease, but to date no experimental study has tested the hypothesis that feeder contamination can directly impact disease status of visiting birds, especially in relation to the population of origin (i.e. urban v. rural, where feeder use/densities naturally vary dramatically). Here we used a field, feeder-cleaning experimental design to show that rural, but not urban, house finches (*Haemorhous mexicanus*) showed increased infection from a common coccidian endoparasite (*Isospora* spp.) when feeders were left uncleaned and that daily cleaning (with diluted bleach solution) over a 5-week period successfully decreased parasite burden. Moreover, this pattern in rural finches was true for males but not females. These experimental results reveal habitat- and sex-specific harmful effects of bird feeder use (i.e. when uncleaned in rural areas). Our study is the first to directly indicate to humans who maintain feeders for granivorous birds that routine cleaning can be critical for ensuring the health and viability of visiting avian species.

## Introduction

Humans impact wildlife in many indirect and direct ways, and these effects can be both positive and negative. Given the recent extent and rate of human expansion across the globe, many are concerned about the range and severity of harmful anthropogenic effects, including pollution, habitat alteration, and climate change^1^. And despite the fact that some human activities may have apparent benefits to either humans or wildlife (e.g. nesting structures for breeding, habitat management, environmental education)^2^, in some cases they carry hidden costs, which may outweigh the benefits.

Supplemental feeding of wildlife may be one such activity^3,4^. Human resource subsidies in gardens and yards provide animals with abundant, predictable foods^5^ and deepen the wildlife knowledge and appreciation of many residents who feed them^6^. However, these food supplies (and the animal aggregations they attract) may also elevate predation risk^7,8^ (including by feral/pet cats in urban areas^9^) or alter nutritional/energy balance^10^. Disease susceptibility is also touted as a putative elevated risk of animal feeding^3,11^, although the mechanism(s) by which this may occur are uncertain^12^. Horizontal disease transmission may increase if feeding stations elevate population density and/or individual-to-individual contact rates^13^. Alternatively, feeders may serve as fomites^14^ (i.e. surfaces/materials that can be contaminated by infection). Animals can leave disease-contaminated materials (e.g. blood, feces, eye discharge) on feeder surfaces that, if not cleaned, can foster and exacerbate transmission of blood-borne pathogens, endoparasites, and other microbes.

This raises the prospects of whether or not extensive soiling of feeders is responsible for elevating incidence or severity of certain pathogens and parasites, and whether or not routine feeder cleaning may be an effective means of reducing disease risk at feeding sites. Hummingbird feeders notoriously require regular sanitization due to sugar spoilage^15^, but outside of mentions in guidelines for feeding birds (e.g. National Wildlife Federation, Project FeederWatch)^16^, data supporting the sanitary value of cleaning wild bird feeders (e.g. seed, fruit, suet) have been virtually absent from the literature to date^17^. Here we undertake, to our knowledge, the first field experiment to determine if feeder soiling or cleaning has direct impacts on avian disease. We performed this experiment with house finches (*Haemorhous mexicanus*), a popular backyard-bird subject for the study of avian disease dynamics^18,19^. Because bird feeders are typically much more concentrated in urban areas^20^, and urban animals can have higher pathogen and parasite burdens^21^ (including in our study species^22^), we also assessed the extent to which feeder soiling or cleaning treatments might differentially impact bird disease in urban v. rural areas. We conducted a 10-week experiment, in which we offset 5-week experimental rotations of active feeder cleaning (i.e. daily with a dilute bleach solution) and soiling (i.e. not cleaned) at an urban study site and a natural desert site and measured changes in a common avian disease—coccidiosis—due to its fecal–oral transmission route on soiled surfaces^22^. We predicted that disease levels would rise during periods when feeders were not cleaned, would fall during sanitization periods, and that, because of comparatively rare presence of bird feeders in rural areas, rural birds may be relatively more impacted by the extent of feeder soiling.

## Materials and methods

We studied house finches at one urban (Arizona State University—Tempe campus) and one rural (South Mountain Regional Park) site. These habitat categorizations were determined previously based on land-use and land-cover attributes as well as human population density within 1 km of the trapping site^22^. In our previous work at these representative sites, we have shown consistent urban–rural phenotypic differences in these finch subpopulations, especially for plumage color expression^23,24^ and disease prevalence/severity^22^, although we recognize that studies at additional urban and rural sites would permit broader urban-ecological generalizations to be made.

On 15 June 2017 (ca. 1 week prior to initial trapping and sampling), we placed 5 plastic yellow 1.8 kg bird feeders (hopper style, Audubon model NA6231) filled with black oil sunflower seeds at each site. Thereafter, we conducted a 10-week “feeder-cleanliness” experiment, which we broke into two 5-week periods of dirty or clean feeders. Specifically, at our urban study site, for the first 5 weeks we cleaned the feeders daily with a 10% bleach solution before rinsing, drying, and re-hanging in the field; in the last 5 weeks of the study, we left the urban feeders uncleaned. At our rural study site, we implemented the same treatments but in the opposite order; we left feeders uncleaned for 5 weeks and then cleaned them as above for the last 5 weeks. We employed this offset rotation to remove any seasonal confounds, although without a true control/reference sample, where we examined seasonal changes in coccidia per se, we cannot rule out the possibility that there were temporal confounds that affect the interpretation of our findings. Feeders were filled every other day; all feeders contained seeds at all times.

We captured finches (from 0500 to 0900 h) using standard walk-in basket traps^25^, banded each bird for individual identification, determined age and sex when possible using plumage characters^26^, measured body mass (to the nearest 0.1 g using an electronic balance), and transported them in small brown paper bags within an air-conditioned vehicle to obtain fecal samples (see below). We trapped on 1–3 days per week, with the aim of capturing up to 10 newly captured (i.e. unbanded) and sexable birds/site/week. Finches were housed in an indoor vivarium at the Arizona State University—Tempe campus upon return from the field site until 1600 h., at which point we began fecal collection (due to the diel cycle of oocyst shedding by *Isospora* spp.^27^). This caging procedure is standard for fecal coccidian analyses in wild birds^22,28^, and it is not expected that very brief captive housing alters parasite oocyst shedding (i.e. time course of coccidia changes is on the order of days^29,30^), although this has not been tested directly in house finches. Birds were housed individually in small wire cages with ad libitum access to sunflower seed and water in the same indoor rooms (kept at 25 °C). At 1600 h we replaced paper at the bottom of the cage with fresh sheets, and at 1700 h, we returned to the room to retrieve the paper and scraped fresh fecal samples from each bird into a solution of 2.2% potassium dichromate^22^. The birds were then placed into their brown paper bags and released at their capture site before sunset. Methods for oocyst isolation and microscopic analyses follow those in Brawner et al.^27^; briefly, we transferred the sample contents into a fresh 9 mL culture tube and filled it to the top with Sheather’s sugar solution (RICCA, Arlington, Texas). We placed a microscope coverslip atop the convex meniscus of the solution on the tube mouth and centrifuged for 5 min at 3000 RPM. We then transferred the microscope slip to a microscope slide, and two scorers examined the slide under a light microscope (Olympus BX60) at × 40 magnification with constant (e.g. equivalent contrast, brightness and hue) microscope settings and ranked oocyst load (i.e. degree of infection) based on a 0–5 scale^27^. We thus had individual-level values of infection (i.e. coccidia scores^31^) that we could use to analyze changes in both the presence and severity (degree of infection) of coccidiosis over time. We did not include birds in this study who showed signs of poxvirus infection, and birds in our study populations do not have mycoplasmal conjunctivitis^32^.

All statistical analyses were run in the R computing environment^33^, with α = 0.05. We developed models that allowed us to test, within each study site: (1) if and how coccidia infections changed over time when birds were held on each of the 5-week clean- or dirty-feeder treatments, and, (2) because of potential sex differences in disease^34^, if and how males and females responded differently to these treatments. We focused on temporal change because our focal prediction was that daily sanitization during the ‘clean’ treatment, or accumulated soiling during the ‘dirty’ treatment, would progressively improve or reduce, respectively, bird health state. Specifically, we built generalized linear models^35^ (with either a binomial error term or, if the data were overdispersed, a quasipoisson error term), separately for our rural and our urban site and for our two measures of coccidiosis (severity and presence), that contained sex, feeder treatment, and time since the feeder treatment began as our main effects and included all possible interaction terms. For the coccidia-presence models, we used logistic regression with a binary response variable (presence/absence of coccidia). We conducted post-hoc tests when variables were statistically significant, with special attention to higher-order terms containing time since treatment began; when a term with this variable was significant (e.g. sex × treatment type × treatment duration), we used either univariate generalized linear models (for coccidia-severity models) or univariate logistic regression (for coccidia-presence models) to test the hypothesis that the slope of the line differed from zero. We also ran Tukey’s post-hoc tests to compare line slopes between the sexes and within the treatments, to determine if disease state changed differently over time when males and females fed from clean v. dirty feeders. We initially included age (hatch-year v. after-hatch-year) as a variable in the models, but no terms containing age (i.e. either as a main effect or in interaction terms) were significant, so we excluded age as a variable in final analyses (Table 1).

**Table 1:** Output of generalized linear models testing effects of sex, feeder treatment, time, and their interactions on coccidia severity and presence in birds from our rural (n = 75 males, 40 females) and urban (n = 62 males, 55 females) study sites.

Response	Site	Predictor	Effect size ± SE	χ^2^	*p*
Coccidia severity	Rural	Sex	2.13 ± 1.45	1.15	0.28
		Treatment	0.60 ± 0.49	0.33	0.56
		Duration	1.01 ± 0.031	0.08	0.77
		Sex × treatment	0.033 ± 0.88	4.46	**0.034**
		Sex × duration	0.93 ± 1.45	3.10	0.078
		Treatment × duration	1.01 ± 0.041	0.03	0.86
		Sex × treatment × duration	1.17 ± 0.07	5.30	**0.021**
	Urban	Sex	1.93 ± 0.87	1.15	0.28
		Treatment	3.55 ± 1.57	4.33	**0.037**
		Duration	1.01 ± 0.019	0.92	0.34
		Sex × treatment	0.16 ± 0.096	4.63	**0.031**
		Sex × duration	0.95 ± 0.025	2.28	0.13
		Treatment × duration	0.95 ± 0.021	4.06	**0.044**
		Sex × treatment × duration	1.10 ± 0.035	5.35	**0.021**
Coccidia presence	Rural	Sex	2.19 ± 3.15	0.29	0.58
		Treatment	1.67 ± 2.74	0.099	0.75
		Duration	1.02 ± 0.077	0.13	0.71
		Sex × treatment	0.015 ± 0.037	3.21	0.073
		Sex × duration	0.93 ± 0.073	1.02	0.31
		Treatment × duration	0.95 ± 0.083	0.27	0.59
		Sex × treatment × duration	1.21 ± 0.14	2.91	0.088
	Urban	Sex	34.12 ± 110.2	1.96	0.16
		Treatment	71.4 ± 243.23	2.48	0.12
		Duration	0.98 ± 0.042	0.071	0.79
		Sex × treatment	0.00019 ± 0.00090	5.74	**0.017**
		Sex × duration	0.83 ± 0.12	2.41	0.12
		Treatment × duration	0.88 ± 0.11	1.48	0.22
		Sex × treatment × duration	1.4 ± 0.26	5.20	**0.023**

We used a quasipoisson error term in models of coccidia severity and a binomial error term in models of coccidia presence. Effect sizes are incident rate ratios for the coccidia severity models, and odds ratios for the coccidia presence models. Degrees of freedom in all tests = 1.

Boldfaced *p*-values indicate significance at α = 0.05.

## Results

### Rural feeder cleanliness and disease

**Figure 1:**
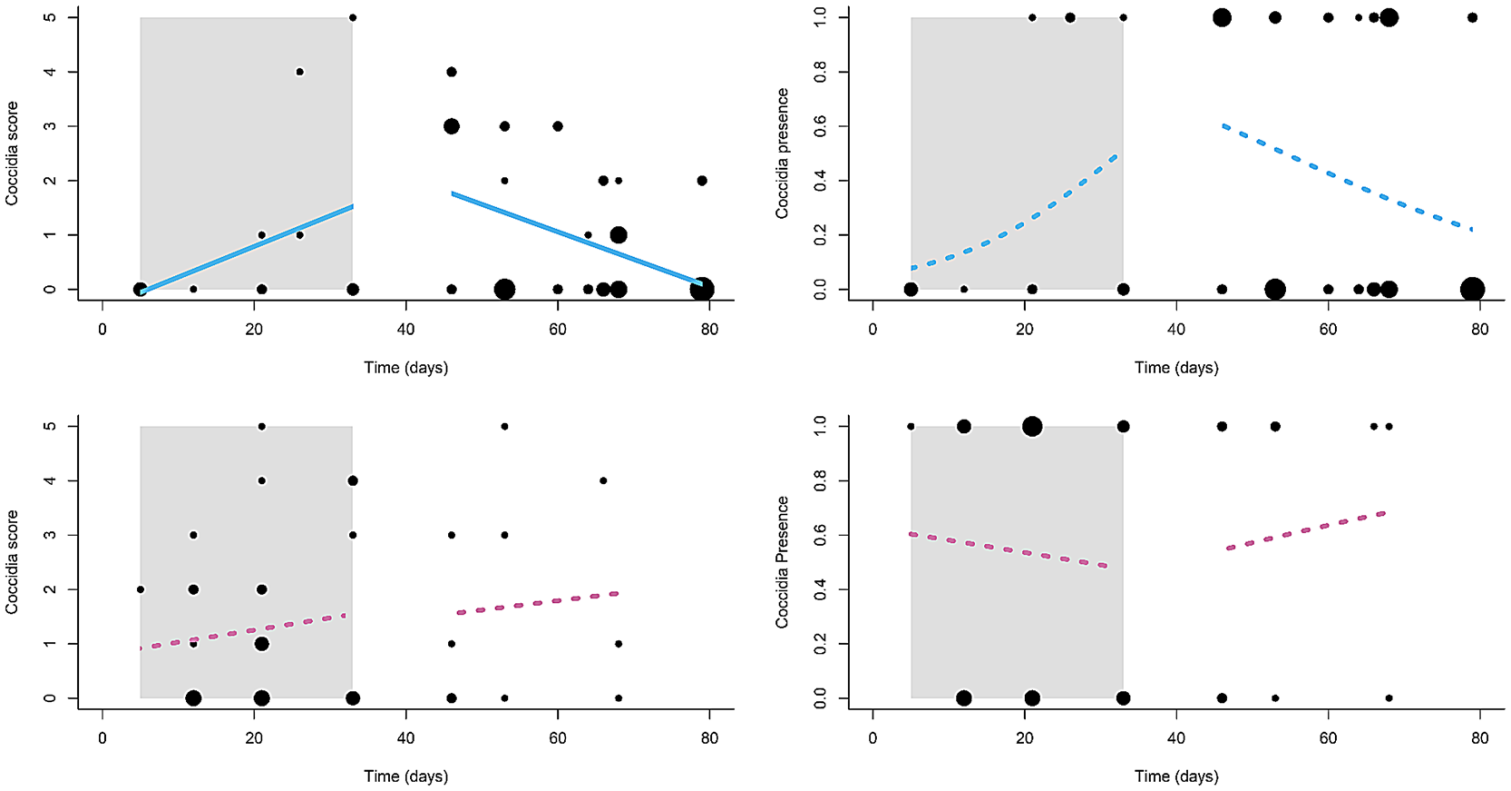
Scatterplots showing temporal variation in coccidiosis in males (blue lines) and females (pink lines) from our *rural* study site on the different feeder treatments (clean vs. dirty): (**a**) coccidia severity (shown as integer scores from 0 to 5), and (**b**) coccidia presence. Dirty-treatment data are shown against a gray background, whereas clean-treatment data are shown against the white background. Point size is proportional to the number of overlapping observations. Solid lines denote slopes that were statistically significant different from zero, whereas dashed lines signify slopes that did not differ from zero. The figure was drawn using R software^33^.

We found significant effects of the sex × treatment and sex × treatment × time interactions on disease severity in rural birds (Table 1). Post-hoc analyses revealed that coccidiosis severity in rural males, but not females, temporally responded differently to the clean v. dirty treatments (Table 2a). Specifically, as predicted, coccidia score in rural males significantly increased over time during the ‘dirty-feeder’ treatment and significantly decreased over time during the ‘clean-feeder’ treatment (Fig. 1, Table 2b); however, severity of coccidiosis did not change significantly over time in response to either feeder-cleanliness treatment in rural females (Table 2b). We found no significant effect of any variable on presence of coccidiosis in rural birds (Fig. 1, Table 1).

**Table 2:** Results of post-hoc analyses comparing disease state between birds of different sex, on the different feeder treatments, and over time within the treatments.

(a) Model type	Site	Contrast	*z*	*p*				
Severity	Rural	Males on dirty v. clean feeders	− 2.75	**0.006**				
		Females on dirty v. clean feeders	− 0.18	0.86				
		Males v. females on clean feeders	1.83	0.067				
		Males v. females on dirty feeders	− 1.35	0.17				
	Urban	Males on dirty v. clean feeders	− 1.36	0.17				
		Females on dirty v. clean feeders	1.99	**0.046**				
		Males v. females on clean feeders	1.50	0.13				
		Males v. females on dirty feeders	− 1.90	0.056				
Presence	Rural	Males on dirty v. clean feeders	–	–				
		Females on dirty v. clean feeders	–	–				
		Males v. females on clean feeders	–	–				
		Males v. females on dirty feeders	–	–				
	Urban	Males on dirty v. clean feeders	1.49	0.14				
		Females on dirty v. clean feeders	− 1.07	0.29				
		Males v. females on clean feeders	− 1.24	0.22				
		Males v. females on dirty feeders	1.40	0.16				

(b) Model type	Site	Feeder treatment	Sex	*n*	β	SE	*z*	*p*
Severity	Rural	Dirty	Male	14	0.10	0.062	4.04	**0.044**
			Female	30	0.017	0.026	0.042	0.51
		Clean	Male	61	− 0.061	0.016	16.18	** < 0.0001**
			Female	10	0.0098	0.041	0.05	0.81
	Urban	Clean	Male	10	− 0.035	0.024	2.06	0.15
			Female	34	0.017	0.018	0.87	0.34
		Dirty	Male	52	0.0091	0.011	0.41	0.52
			Female	21	− 0.034	0.015	4.89	**0.027**
Presence	Rural	Dirty	Male	14				
			Female	30				
		Clean	Male	61				
			Female	10				
	Urban	Clean	Male	10	− 0.19	0.14	− 1.36	0.17
			Female	34	− 0.11	0.042	− 0.26	0.79
		Dirty	Male	52	0.023	0.033	0.70	0.49
			Female	21	− 0.13	0.11	− 1.25	0.21

(a) Pairwise Tukey tests of line-slope differences between groups (e.g. sexes, feeder treatments), for both our coccidia-severity and -presence models.

(b) Univariate tests of line-slope differences from zero, for both our coccidia-severity and -presence models. Severity model results are again based on quasipoisson error terms when data were overdispersed. Rows containing dashes/lines instead of data represent models for which we found no statistically significant predictors (i.e. post-hoc tests are not appropriate).

Boldfaced *p*-values indicate significance at α = 0.05.

### Urban feeder cleanliness and disease

**Figure 2:**
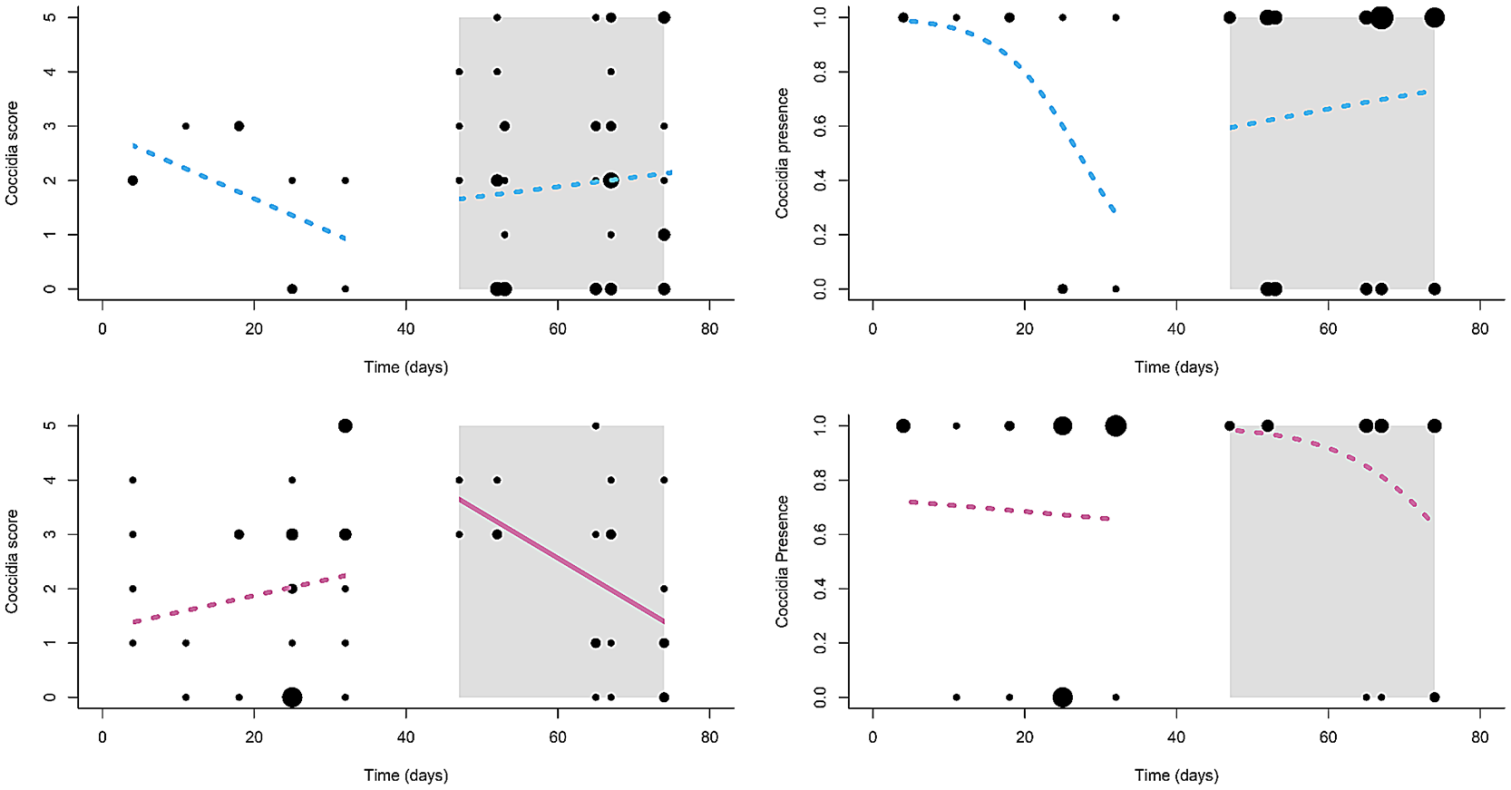
Scatterplots showing temporal variation in coccidiosis in males (blue lines) and females (pink lines) from our *urban* study site on the different feeder treatments (clean vs. dirty): (**a**) coccidia severity (shown as integer scores from 0 to 5), and (**b**) coccidia presence. Dirty-treatment data are shown inside the gray boxes, whereas clean-treatment data are shown against the white background. Point size is proportional to the number of overlapping observations. Solid lines denote slopes that were statistically significant different from zero, whereas dashed lines signify slopes that did not differ from zero. The figure was drawn using R software^33^.

We found significant effects of four terms—treatment, and the sex × treatment, treatment × time, and sex × treatment × time interactions—on coccidiosis severity in urban birds (Table 1). Specifically, severity was highest when feeders were not cleaned, and temporal change in coccidiosis score differed for females, but not males, when they fed from clean v. dirty feeders (Table 2a). Interestingly, neither males nor females became healthier on the ‘clean-feeder’ treatment, and coccidiosis severity in males also was unaffected by the ‘dirty-feeder’ treatment (Fig. 2, Table 2b). However, contrary to our prediction, female coccidiosis severity declined on the ‘dirty-feeder’ treatment (Fig. 2, Table 2b).

We also found significant effects of the sex × treatment and sex × treatment × time interactions on disease presence in urban birds (Table 1). The slopes of the four lines (i.e. temporal change in disease presence for males and females on each of the dirty and clean-feeder treatments) did not differ significantly from zero, however, indicating no clear response by either sex to either the clean- or the dirty-feeder treatment (Fig. 2, Table 2b). Post-hoc Tukey tests also revealed that none of the line slopes differed from one another (all *p* > 0.14), which is likely due to adjustments for family-wide error rates.

## Discussion

We performed the first field experimental test of the effectiveness of bird-feeder cleaning on avian disease transmission. Using a widespread backyard bird species from North America (the house finch), we also considered differential effects of feeding cleanliness on disease in urban v. rural birds. Based on the premise that, among many other traits^23,36,37^, urban and rural birds differ significantly in their exposure to and use of supplemental feeders, we predicted that rural birds would be comparatively more impacted by the introduction of soiled and cleaned feeders. We found intriguing habitat- and sex-specific effects of a daily bleach feeder cleaning treatment on coccidiosis in house finches. Namely, rural males became significantly more infected with endoparasitic coccidians when feeding from uncleaned feeders and significantly less infected when feeding from clean feeders; however, we found no such effects in urban males or in females from either site. Overall, these results support the health-beneficial effects of routine bird feeder cleaning but only in a specific cohort of birds (i.e. males from rural areas).

There are many literature suggestions about the value of cleaning bird feeders, but none to date have come with rigorous experimental support. For example, extensive work on mycoplasmal conjunctivitis in house finches has shown that birds who visit feeders are more likely to be infected^11,38^ and that feeders themselves can serve as active fomites for disease transmission (i.e. by allowing diseased birds to feed from feeders and showing that birds who subsequently feed from those feeders can contract conjunctivitis without direct contact with infectious birds, thus actively contaminating the feeders^14^). Also, various organizations (e.g. Humane Society, Wild Birds Unlimited, Cornell Laboratory of Ornithology) recommend on their websites that feeders be cleaned in some fashion (i.e. with water, bleach, scrub, or some combination) and with frequencies ranging from ‘regularly’ to biweekly. However, it has largely been unclear how effective these methods are for any particular infectious agent, bird species, sex or age class, or feeder surface/location. To our knowledge, there are only two studies that have tested effectiveness of bird feeder cleaning methods on microbial communities. Boyd et al.^39^ found that 10% bleach wipes reduced numbers of aerobic, but not gram-negative, bacterial colonies on feeder surfaces, but that such cleaning had little impact on rates of seed consumed by birds from those feeders (with one unusual exception, that *more* food was consumed when more gram-negative bacteria were present). Feliciano et al.^17^ found that several forms of single-instance cleaning (e.g. bleach, soap/water, scrub) were effective at reducing *Salmonella* colonies on feeders, but that more thorough cleaning procedures (e.g. bleach and scrub; not soap/water) are needed when debris has accumulated on a feeder. However, in both instances, the efficacy of the cleaning methods was not directly tested by monitoring disease state in feeder-visiting birds. Here we used a daily bleach-scrub and water-rinse protocol to clean feeders and effectively reduce gut coccidian loads in rural male house finches. We chose to use this frequent, more liberal method in order to ensure daily cleanliness and broad anti-microbial effectiveness, but it will be interesting now to see if different (e.g. other cleaning products, without scrubbing) or more conservative (i.e. weekly) procedures could also be effective in combating disease in feeder-visiting birds.

The fact that the effect of feeder cleaning on disease was limited to just rural males was surprising. In fact, in one instance, we found that urban females had significantly *lower* coccidia levels as they fed more from a soiled feeder. Why might disease state in the two sexes have responded differently to feeder cleanliness? We originally suspected a stronger effect of our treatments in comparatively naïve rural birds (i.e. which may not commonly feed from feeders and that permit them to aggregate in high density), but one idea for why females were not affected may be attributed to female social dominance in this species. Female house finches displace males at feeders^40^ and thus presumably gain access to preferred feeding locations. Moreover, because house finches from their native range show significant aversions to feeding near sick conspecifics^41^, it is possible that females also choose to feed at feeder microsites that reduce their disease risk (i.e. avoid standing in/near fecal deposits containing coccidian oocysts). This would leave them comparatively less impacted by feeder cleaning efforts, and instead males would be more likely to be left to feed on riskier, soiled surfaces. It will be interesting now to test whether females do in fact show such clean-microsite feeding preferences (and more so among urban birds, which have greater familiarity/history with feeders, than rural birds).

An alternative to this environmental/behavioral explanation is one centered on physiological/immunological differences. In prior work on these finch populations, researchers have shown that males are consistently more colorful and healthier (including having less coccidiosis) in rural compared to urban areas^22–24^. However, it was recently found that urban female house finches are *less* severely infected by coccidia than rural females^42^. This opposing pattern between males and females suggests complex disease dynamics in urban areas (i.e. involving different or more diverse transmission methods, interactions with other pathogens or parasites), such that urban females do not necessarily spread or acquire more coccidia from feeders simply because, compared to rural birds, they feed more from feeders. Immune responsiveness in songbirds, for example, can differ between sexes^43^ and in relation to urbanization^44^. Last, because a portion of our experimental study overlapped with the late breeding season of house finches in Arizona and because we did not conduct routine feeder observations of bird visits, we also cannot rule out the possibility that males and females visited feeders differently (and were thus differentially impacted by our cleaning treatments) due to their separate breeding roles. For example, male house finches provide greater nutritional provisions to older nestlings and fledglings^45^, including accompanying them at feeders (K.J.M, pers. obs.). Surprisingly, to date, there is little information across feeder-visiting bird species whether the sexes differ significantly in feeder use, but consistent with this some have found (including in house finches) that males tend to visit feeders more than females^46^, although females tend to have longer visitation bouts^47^. This now seems like a profitable avenue to pursue, both as it relates to our disease findings here but also generally to improve our understanding of the sex-specific foraging ecology, nutritional physiology, and even predation risks^7^ of feeder-visiting species.

In sum, we show conditional (specific to males from a rural area) field experimental evidence that feeder soiling and cleaning can have significant impacts on endoparasitism in a common desert and urban passerine species. This result is consistent with suggestions from prior studies on feeder transmission of a different bacterial disease (mycoplasmosis) in house finches, such that feeder surfaces themselves can play a key role in disease transmission^14^. Our study also has broad implications for wildlife health and management, where bird feeder cleaning has been recommended by many but without clear guidance on protocols or expected effects. We uncovered no evidence for benefits of feeder cleaning in urban areas, suggesting possible adaptive co-evolution of birds with feeders and feeder-transmitted disease and that feeder cleaning here may not be as critical, although further work is needed to broadly test this idea among cities, species, seasons, and perhaps including community-wide feeder-cleaning intiatives. We urge others to also consider this conservation practice as it relates to their wild animal model, the pertinent diseases, and other transmission-risk factors, such as feeder-surface type, geographic location, and time of year.
